# Glandular Morphometrics for Objective Grading of Colorectal Adenocarcinoma Histology Images

**DOI:** 10.1038/s41598-017-16516-w

**Published:** 2017-12-04

**Authors:** Ruqayya Awan, Korsuk Sirinukunwattana, David Epstein, Samuel Jefferyes, Uvais Qidwai, Zia Aftab, Imaad Mujeeb, David Snead, Nasir Rajpoot

**Affiliations:** 1 Department of Computer Science and Engineering, Qatar University, Doha, Qatar; 2 Department of Computer Science, University of Warwick, Coventry, UK; 3 Mathematics Institute, University of Warwick, Coventry, UK; 4 Hamad Medical Corporation, Doha, Qatar; 5 Department of Pathology, University Hospitals Coventry and Warwickshire, Coventry, UK

## Abstract

Determining the grade of colon cancer from tissue slides is a routine part of the pathological analysis. In the case of colorectal adenocarcinoma (CRA), grading is partly determined by morphology and degree of formation of glandular structures. Achieving consistency between pathologists is difficult due to the subjective nature of grading assessment. An objective grading using computer algorithms will be more consistent, and will be able to analyse images in more detail. In this paper, we measure the shape of glands with a novel metric that we call the Best Alignment Metric (BAM). We show a strong correlation between a novel measure of glandular shape and grade of the tumour. We used shape specific parameters to perform a two-class classification of images into normal or cancerous tissue and a three-class classification into normal, low grade cancer, and high grade cancer. The task of detecting gland boundaries, which is a prerequisite of shape-based analysis, was carried out using a deep convolutional neural network designed for segmentation of glandular structures. A support vector machine (SVM) classifier was trained using shape features derived from BAM. Through cross-validation, we achieved an accuracy of 97% for the two-class and 91% for three-class classification.

## Introduction

**Figure 1:**
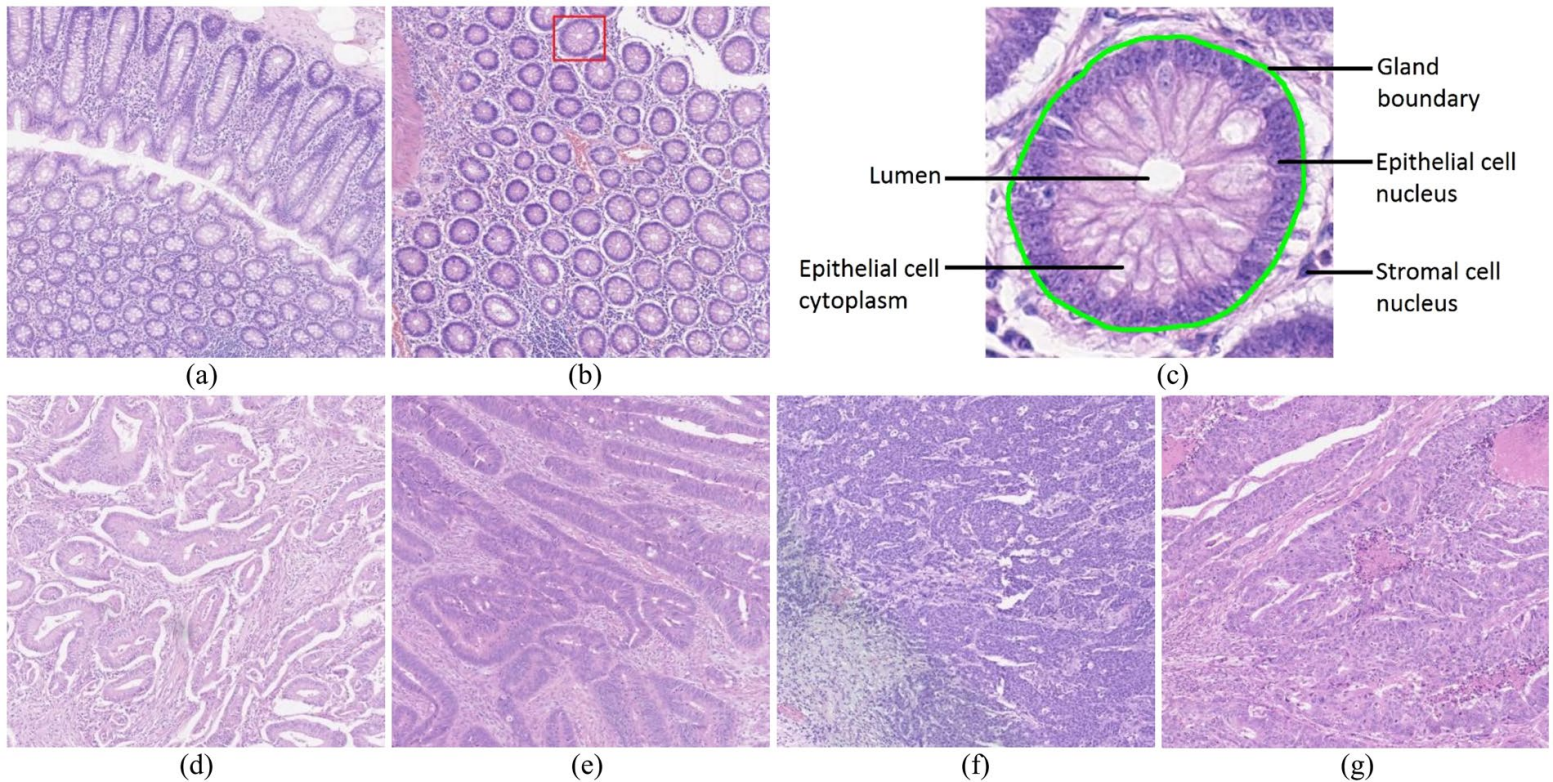
Example images of different grades of tumour. (**a**) and (**b**) show images of normal tissue, (**c**) shows the structure of a normal gland taken from (**b**) (red rectangle in (**b**)), (**d**) and (**e**) show low grade tumour images while (**f**) and (**g**) show high grade tumour images.

Colorectal cancer is one of the most common cancers and the fourth most common cause of cancer related deaths^1^. In 2012, it accounted for approximately 10% of the cancer cases recorded worldwide and was the third and second most common cancer in men and women respectively^1^. Adenocarcinomas originate from epithelial cells^2^ and account for over 90% of colorectal tumours. Determining the grade of cancer from colorectal tissue slides is a routine part of the pathological analysis and is one of the potentially useful parameters for deciding the treatment plan. Grading is currently carried out on the basis of the degree of glandular differentiation/formation which a tumour shows. For example well-differentiated tumours are predominantly glandular (see Fig. 1(d) and (e)) while in poorly differentiated tumours, the epithelial cells forming the gland boundary will diffuse irregularly, making it challenging to locate the boundary of individual glands (see Fig. 1(f) and (g)). The structure of a normal gland is shown in Fig. 1(c). Glands, also termed *tubules* or *crypts* in the literature, are three-dimensional testtube-like structures. Normal glands occur in a well-organised fashion, whose appearance, after slicing through the tube to obtain a tissue section, is elliptical, or possibly circular, depending on the angle that the section makes to the tube. High grade adenocarcinomas, however, show large variations in the degree of gland formation as well as in the morphology of these glands.

Achieving consistency between pathologists is difficult due to the subjective nature of grading assessment and the fact that many tumours show varying patterns of differentiation. In addition to inter- and intra-observer differences, this process is time-consuming for pathologists. Despite this variability, tumour grade has been shown to have clinical and prognostic significance^2–5^. Currently, there is no accepted standard grading system. However, most of the systems stratify tumours into four grades (or combinations of them): grade 1 (well differentiated), grade 2 (moderately differentiated), grade 3 (poorly differentiated) and grade 4 (undifferentiated). In most of the studies focusing on the prognostic importance of grading, several grades have been merged to form a two-tiered grading system as follows: the combination of well and moderately differentiated (grades 1 and 2) is defined as *low grade*, while the combination of poorly differentiated and undifferentiated (grades 3 and 4) is defined as *high grade*. This system retains prognostic significance, while reducing inter-observer variability^3,6^.

Stratification of a tumour into low and high grade CRA is recommended to be based solely on the degree of gland formation^4^. A widely used criterion for the two-tiered system is to classify a tumour as low grade if 50% or more of a tumour is glandular, and as high grade if the percentage is less than 50%. Figure 1 shows some example images of normal tissue, low grade tumour and high grade tumour. Although this grading scheme has reduced inter-observer variability, it is still based on subjective assessment. In order to avoid subjectivity and to make assessment fast, there is an increasing demand for an objective computer-aided measure that can assist pathologists with a two-tiered grading system.

The introduction of digital whole slide images (WSIs) has led to the possibility of using digital slides for analysis on a regular basis, with some laboratories opting to go fully digital. This means that computer algorithms that can objectively measure tumour grade are likely to be used in future to improve the accuracy and consistency of grading and extend the potential of tumour grading to be an effective tool for management decision. This is particularly relevant to CRA due to the subjective nature of assessment^6^. In recent years, researchers have proposed several computational methods that provide objective measures for grading of breast and prostate cancer^7–9^. The non-trivial step in automatic tumour grading based on the morphology of glands is gland segmentation. Automated segmentation of glands is a challenging task because of the variation in tissue architecture. This variability has various sources, including differences in staining, methods of slide preparation, time elapsed between slide preparation and scanning of the slide, and differences between different brands or models of scanners from different manufacturers. And, of course, there is also biological variability.

Shape based features such as size and roundness are known to be useful in separating normal tissue from a tumour^7^. Here we extend this work, measuring the extent to which the shape of a gland in a possible tumour region differs from the shape of a gland in a healthy region. To be more precise, a gland is 3-dimensional, while an image is 2-dimensional. An image contains pictures of the ways in which a plane cuts through various glands at various positions and orientations. Healthy glands (remember that here ‘gland’ means ‘crypt’) have a testtube-like shape. Therefore a micrograph shows a healthy gland as bounded by an approximate circle or ellipse. (Occasionally one sees the open end of the crypt where it joins the intestine; however, our gland segmentation program does not classify open ends of crypts as glands, so such structures are ignored.) Such circles and ellipses can be of very different sizes and shapes, even when the corresponding glands are all approximate testtubes of almost the same 3-dimensional shape.

We will use the ‘distance’ between shapes of glands, more technically, a *metric on shape space*. In a micrograph of a 2-dimensional tissue section, the shape of a gland is captured by its 1-dimensional boundary, which is almost always a simple closed curve. Since a section of a biopsy has no ‘up’, ‘down’, ‘left’ or ‘right’, and since the curve of interest is a subset of the plane, rather than a parametrised curve, the metric on shape space needs to be invariant under translation, rotation and change of parametrisation. Several metrics on shape space have been investigated^10^. We use BAM (Best Alignment Metric) on the space of shapes^11^ because of its fast computation and also because of other good properties which are demonstrated with experimental results in the Supplementary Materials document.

The main focus of this study is to show that one can identify the degree of differentiation of a colon tissue image by computer analysis of the shape of glands. The shape of glands is also a main criterion used for the same purpose by pathologists. We have experimented extensively to demonstrate a strong correlation between the grade of a tumour and the degree of deviation of the shape of its glandular structures from the normal elliptical/circular shapes. This relation can be used to identify images showing normal tissue, low grade tumour or high grade tumour. We used a pixel-based deep neural network for gland segmentation^12^ and trained it on digitised images of tissue slides stained with Haematoxylin & Eosin (H&E). We modified this network in order to improve the segmentation results. To measure the glandular aberrance quantitatively, we then computed BAM values, using the network output to identify gland boundaries, and analysed its correlation with the various grades of the cancerous tumour.

## Previous Work on Automatic Grade Determination and Gland Segmentation

Naik *et al*.^8^ used morphological features representing the shape and size of glands to distinguish between different grades of prostate cancer. They performed three two-class classifications: (a) normal vs grade 3, (b) normal vs grade 4 and (c) grade 3 vs grade 4. For gland segmentation, they presented a model that incorporates low, high and domain level information. A Bayesian classifier is used to classify the lumen, stroma and nuclei, and then, using domain level knowledge, the true lumen is identified. This is further used to initialise a level set contour for gland boundary segmentation. Farjam *et al*.^7^ formulated a cancer index based on the size and shape of glands, which corresponds to the malignancy of cancer, and serves to differentiate between normal tissue and malignant prostate cancer. Nguyen *et al*.^9^ performed three-class classification into normal, Gleason grade 3 and Gleason grade 4 patterns of prostate cancer.

The reliability of any statistical measure, indicating the aggressiveness of a tumour on the basis of gland morphology, depends on how accurately the glands are segmented. Existing methods for gland segmentation can be classified into two broad categories: (a) hand-crafted feature-based approaches and (b) deep neural network methods. We have used the latter approach. All the works on cancer grading mentioned in the paragraph above use hand-crafted features. Gunduz-Demir *et al*.^13^ proposed an object-graph based method which represents each component of tissue (lumen and nucleus) as a graph vertex, with edges connecting nearby vertices. This method uses lumen falling inside the gland as an initial gland seed for region growing. Epithelial nuclei lying at the gland boundary serve as a stopping criterion for region growing. All the above mentioned approaches and other approaches^14,15^ have limitations. For example, some rely on pixel level information (colour and texture) and consider the architectural regularity of components of gland where the nuclei appear prominently at the gland borders surrounding the cytoplasm and lumen. Therefore, the performance of these methods is liable to be affected by stain variation and irregularities of glands in colon tumours. In view of the limitations of above methods, Sirinukunwattana *et al*.^16^ proposed a Random Polygons Model (RPM) which segments glands successfully in both normal and poorly differentiated samples. However it is capable of producing slightly different segmentation results from the same image due to its stochastic nature.

Deep Neural Networks are known to produce state-of-the-art results for a number of problems, such as image recognition, voice recognition, object segmentation, hand-writing recognition etc. Such networks have been increasingly applied to medical image processing^17^, specifically in histopathology for mitotic cell detection^18,19^ and classification^20^, tumour segmentation^21,22^, blood cell counting^23^ and gland segmentation^24–26^. Chen *et al*.^25^ proposed a contour aware deep learning architecture for glandular segmentation that integrates the gland object and its contour into a single network. Kainz *et al*.^24^ employed two convolutional neural networks inspired by a classical LeNet-5 architecture, one for detecting the glandular object and the other for separating clustered glands. Janowczyk *et al*.^27^ have investigated the CIFAR-10 AlexNet network architecture for various uses in digital pathology, including gland segmentation. BenTaieb *et al*.^26^ proposed a multi-loss convolution network that performs both classification and segmentation of adenocarcinoma glands.

## Results

The SVM classifier was employed to perform (1) normal vs cancer and (2) normal vs low grade vs high grade classification using Feature Sets 1 and 2. Feature set 1 includes the average BAM value and BAM entropy and Feature set 2 comprises Regularity Index and Feature set 1. The classification accuracies are shown in Table 1) with and without feature postprocessing. The accuracy mentioned in the table represents the percentage of the total number of images that are classified correctly. Feature postprocessing involves removal of BAM values of tangential sections of crypts and glands at image borders from the images of normal classified tissue only. We excluded the BAM values for these glands, since those BAM values were artificially high (details of the method are given under heading’Feature Postprocessing’ in the ‘Glandular Aberrance Features’ subsection). Our results demonstrate that the classification accuracy improves after postprocessing.

**Table 1:** Classification accuracy with and without feature postprocessing. Feature Set 1 comprises average BAM value and BAM entropy while Feature Set 2 comprises Regularity Index and Feature Set 1. Regularity Index is the ratio of the number of glands in the first two bins of the histogram of BAM-values to the total number of glands in an image—see the histograms in Fig. 2. Here accuracy refers to the percentage of number of images classified correctly. The reported values are *mean* ± *standard deviation*, where the variation is the result of cross-validation runs.

	Before Feature Postprocessing		After Feature Postprocessing	
	Feature Set 1	Feature Set 2	Feature Set 1	Feature Set 2
Normal vs Cancer	94.25 ± 1.22	94.97 ± 1.17	95.70 ± 2.10	97.12 ± 1.27
Normal vs Low grade vs High grade	85.63 ± 3.16	88.53 ± 5.28	87.79 ± 2.32	90.66 ± 2.45

Using Feature Set 2, the classifier (SVM) accuracies before feature postprocessing were improved by 3% for normal vs low grade vs high grade cancer. After thresholding, the accuracies were improved by 2% and 3% respectively. These results are presented in Table 1. Our experiments show promising results for classifying the images into normal tissue vs cancer with feature set 2, after postprocessing. However, classification of normal vs low grade vs high grade requires improvement. In Table 2, accuracy, precision, recall and F1-score are presented for two other categories: Cancer and High Grade. In the row labelled ‘Cancer’, both low and high grade samples are regarded as positive. In the row labelled ‘High grade’, both normal and low grade are regarded as negative.

**Table 2:** Evaluation results of 3-class classification, using feature set 2. The accuracy, precision, recall, specificity and F1-score obtained on the test dataset are shown for two categories: Cancer and High grade. We have considered both low and high grade as positive classes in Cancer category while in High grade category, normal and low grade are considered as negative class. Here accuracy refers to the percentage of sum of true positives and true negatives. The reported values are *mean* ± *standard deviation*.

Class	Accuracy	Precision	Recall	Specificity	F1-score
High grade	92.83 ± 3.19	84.08 ± 11.72	91.16 ± 9.10	93.31 ± 6.57	86.71 ± 4.54
Cancer	97.83 ± 2.17	97.10 ± 2.52	98.48 ± 2.62	97.16 ± 2.46	97.78 ± 2.27
Average	95.33 ± 2.68	90.59 ± 7.12	94.82 ± 5.86	95.24 ± 4.52	92.25 ± 3.41

**Figure 2:**
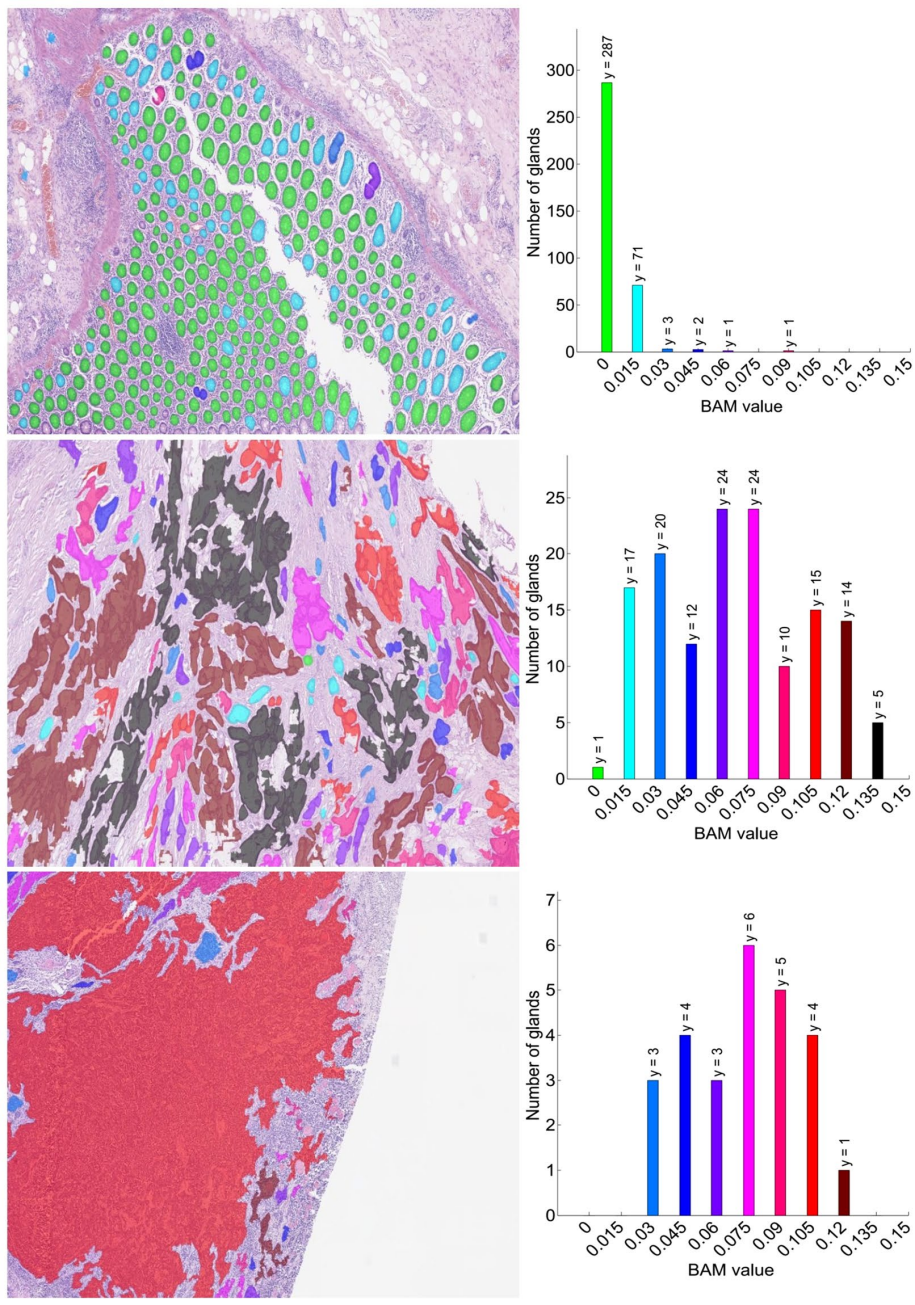
Example of original images overlaid with the gland segmentation mask along with their BAM histograms. Each glandular object is coloured by the colour of histogram bin to which its BAM value belongs. Upper, middle and bottom rows show the overlaid images of normal tissue, low grade tumour and high grade tumour images respectively along with their histograms of BAM values on the right. The *y* value at the top of each bar indicates the number of glands in each bin.

**Figure 3:**
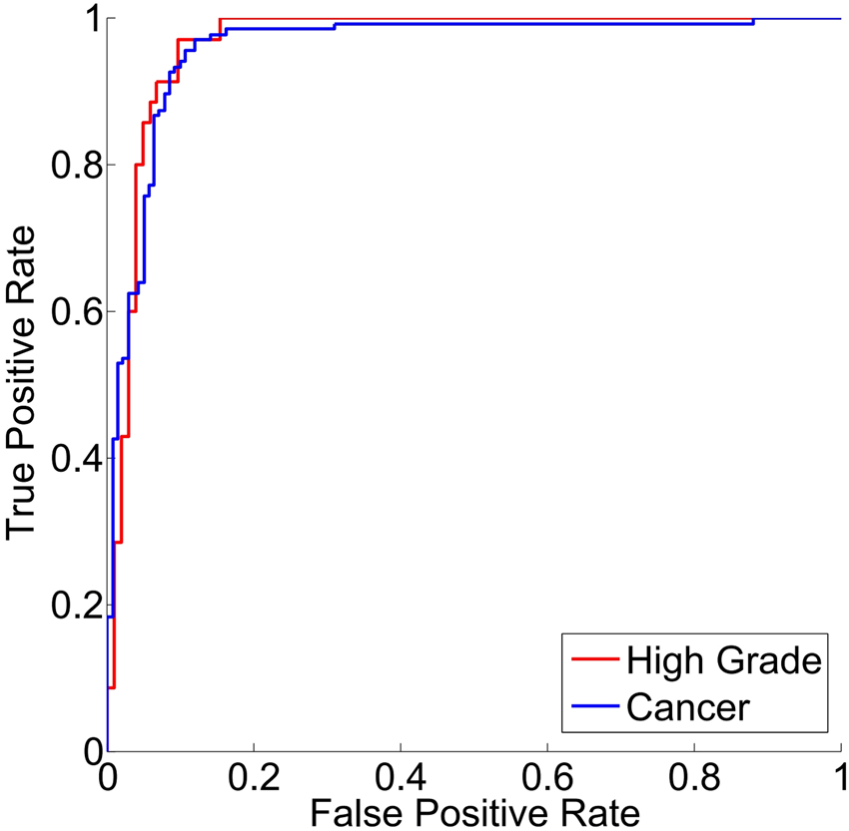
ROC curves. Note that the cancer class comprises low grade and high grade cancer.

Figure 3 shows the receiver operating characteristic (ROC) curves for high grade tumour and cancer (low grade and high grade tumour). It shows more area under curve (AUC) for high grade tumour than for cancer. For qualitative analysis of BAM distance values, one example image for each grade is shown in Fig. 2 along with the histogram distribution of BAM values of all the glands in the corresponding image. Each bin is assigned a different colour. The segmentation mask is then overlaid on the original image with each gland represented by the colour of the histogram bin to which its BAM value belongs. It can be observed that in the case of normal images, more glands lie in the bins of small BAM values. As the tumour progresses to low and then high grade, the gland moves to bins containing larger BAM values.

**Figure 4:**
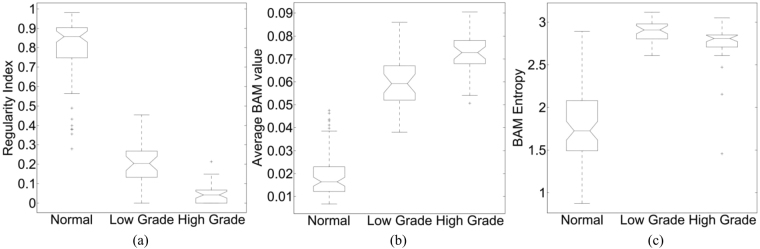
Box plots of (**a**) Ratio of the frequency of first two bins of BAM to the overall frequency, (**b**) Average BAM and (**c**) BAM entropy. The box plots are generated after feature postprocessing. Our classification into three distinct populations (normal, low grade and high grade) has *p*-value less than 0.001 for each of the three criteria (Regularity Index, average BAM value and BAM entropy).

To test whether the samples of statistics for average BAM values and entropy of BAM came from the same populations, the Kruskal-Wallis test was performed. All p-values were less than 0.001. Boxplots of BAM features after feature postprocessing are shown in Fig. 4. Boxplots of BAM features before feature postprocessing are shown in Supplementary Fig. 3 in the Supplementary Materials document. An overlap can be seen between low grade and high grade tumour making three-class classification challenging.

## Discussion

We have performed statistical analysis to show that glandular aberrance is strongly associated with the degree of differentiation of a tumour. Our results demonstrate that the BAM features can be used to distinguish cancerous CRA tissue from normal colorectal tissue for further analysis. We would like to re-emphasize that 3-fold cross-validation was applied in our experiments due to the limited size annotated dataset. The validation on this dataset gave promising results which would support a proposal for a larger validation study.

A merit of BAM is that it is fast to compute, as compared to most other metrics on shape space. Moreover, the mathematics required to understand BAM is simple and easy to grasp, whereas most other methods use advanced mathematical ideas — typically, Riemannian geometry on an infinite dimensional manifold. For time comparisons between BAM and other metrics on shape space, the reader is referred to the Supplementary Materials document.

The efficacy of BAM features in identifying the tumour grade is highly dependent on the accuracy of gland segmentation. The segmentation method should be able to generate a map in which each connected object represents an individual gland, particularly in the case of normal images. If two normal glands are mistakenly merged, a large BAM value may result, possibly leading to a misclassification. In order to evaluate our gland segmentation results, we used a dataset^28^ for which the clinical ground truth was provided. The test dataset was provided in two sets: Part A and Part B with 60 and 20 images respectively. We generated a segmentation mask for the test dataset using our trained network and compared our results with the top four participants of the GlaS challenge^28^. For comparison, we used the same criteria for evaluation, as those used by the challenge organizers. The evaluation results are shown in Supplementary Table 3. We also evaluated our results for benign and malignant test images separately and found that our approach performed better than the challenge winners for malignant images, as shown in Supplementary Fig. 4 in the Supplementary Materials document. Although, we performed segmentation postprocessing to handle most of the artifacts, the improvement is still needed particularly for segmentation of normal/benign glandular structures.

For comparison purpose, we have presented results for other standard and potentially relevant morphological features (such as roundness, aspect ratio, elongation, solidity and convexity) in Supplementary Fig. 5. Although these features are fast to compute as compared to the computation of BAM features but they do not perform better in terms of classification accuracy. As can be seen in the Supplementary Fig. 5, the BAM features perform better when compared with any combination of these standard shape features, for both 2-class and 3-class classification.

A single WSI can include tumour regions of more than one grade. Hence we assigned a grade to images of selected visual fields from WSIs. As we know now that features based on the BAM measure are highly correlated to grade (more specifically, to the distinction between normal and cancer), the same operation can be performed for a WSI by considering images of suitable size in a sliding window fashion and then calculating the average BAM distance and relevant BAM features to grade them. Such an approach could offer an automated solution; downstream of the pathologist’s initial assessment. For example, on the basis of looking at a few patches, the pathologist might identify a particular slide on which grading should be done. The algorithm would then assess the grade across the entire slide. Future work will include lumen features to improve the results of three-class classification. We will also add assessments of other parameters of grade, such as nuclear morphology, mitotic rates and extent of necrosis. This opens up the possibility of automated quantification of different grades within a single tumour. Such results could be tested for their relevance in prognostics. Such an approach would hardly be possible with an unassisted visual grading of the tumour.

## Materials and Methods

The tissue slide images and associated clinical data were obtained from the University Hospitals Coventry and Warwickshire (UHCW) NHS Trust in Coventry, UK. The data used for this study including the WSIs and grading information was provided after de-identification and informed patient consent was obtained from all subjects. Ethics approval for this study was obtained from the National Research Ethics Service North West (REC reference 15/NW/0843). All the experiments were carried out in accordance with approved guidelines and regulations. Data and code used for this study will be made available if the paper is accepted.

For this study, we used digitised WSIs of 38 CRA tissue slides stained with H&E. All WSIs were taken from different patients and were scanned using the Omnyx VL120 scanner at 0.275 *μ*m/pixel. From each WSI, a number of non-overlapping images of size 4,548× 7,548 pixels were extracted at magnification 20× and were labelled as normal tissue, low grade tumours or high grade tumours by an expert pathologist. In total 139 images were extracted, comprising 71 normal, 33 low grade and 35 high grade cancer images. Using this dataset, we looked for a separation line between normal and tumour, and for a way of discriminating between normal, low grade and high grade cancer, using BAM features. We evaluated the performance of BAM features in classifying the tumour into different grades by 3-fold cross validation. Splitting of image data for training and testing phase was performed on extracted images rather than on the patient level. This means that, given two images from a single WSI, one image may be used for training and the other for testing, or both images may be used in the same phase. However, there was no overlapping of images, and each region was labelled independently. This data split could not be performed at the patient level due to the class imbalance problem. For gland segmentation, we initially used another dataset^29^, comprising 37 normal and 48 tumour images, for training the network. Looking at the variations in a glandular structure in tumour images of our dataset, it became clear that the collection of images in the dataset^29^ was too small for effective training. We therefore extracted additional images from our WSIs, annotated them and added them to our training set.

**Figure 5:**
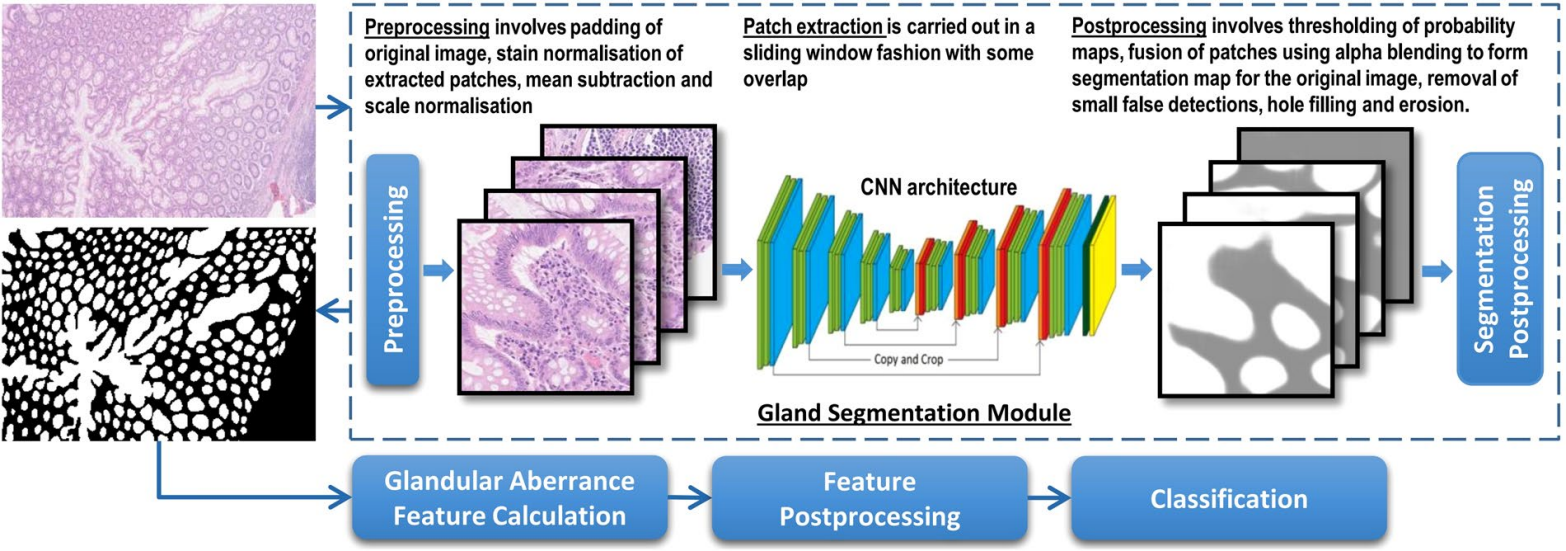
An overall flowchart of our methodology.

In a micrograph of a tissue section from a biopsy of a suspected colon cancer, we first locate the simple closed curves that are boundaries of the various glands. To each such simple closed curve *γ*, we associate a ‘best’ approximating ellipse *α*, and then compute the BAM distance between *α* and *γ*. We define this distance to be the *aberrance* or *BAM value* of *γ*. A detailed description of locating the gland boundaries and computation of the corresponding BAM values is given below. A block diagram to show the overall flow of our methodology is presented in Fig. 5.

### Gland Segmentation

**Figure 6:**
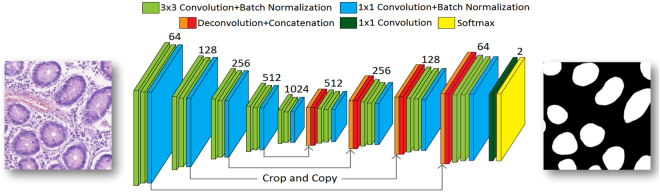
Network architecture. Each slice represents a multi-channel feature map and the depth of each feature map is mentioned on the top of layers. The orange slice is the copied feature map cropped from the corresponding down-sampling layer.

For gland segmentation, we employed a convolutional neural network (CNN) architecture based on a modified version of Ronneberger *et al*.’s^12^ UNET architecture. This network is modelled to perform pixel-based classification, taking an image as input and output an image of the same or smaller size depending on the type of convolution used in the network. Each pixel in the output image represents the probability of the respective pixel in the input image belonging to a glandular structure. This probability map is then further processed (e.g., by simple thresholding) to produce a segmentation mask for the input image. An overview of the modified architecture of the training network is shown in Fig. 6. In order to improve the performance, we made the following changes to the original UNET architecture: 1) addition of a batch normalisation^30^ operation, 2) removal of the dropout layer, 3) addition of a 1 × 1 convolution operation in each layer 4) use of Adadelta as the optimisation strategy instead of stochastic gradient with momentum and 5) weighted cross entropy as an objective function.

#### Patch Generation for Training and Testing

During the training phase, our network was provided with non-overlapping RGB input patches of size 428 × 428 pixels along with their ground truth mask. In order to perform effective training, augmentation was performed using flip, rotation and elastic distortion. During the inference phase, overlapping patches of size 428 × 428 pixels were extracted from our dataset comprising images of size 4548 × 7548 pixels. The output probability maps of these patches were merged to generate segmentation masks for our dataset. During the inference phase, some artefacts were observed around the patch. In order to avoid such artefacts, we extracted all the test patches with 25% extra overlap. The output maps of the overlapped patches were merged together via alpha blending to generate the segmentation mask. Due to alpha blending, the resulting segmentation mask was relatively smooth. More details on training the network can be found in the Supplementary Materials document.

#### Preprocessing

In digital histopathology images, colour inconsistency becomes a significant issue in the autonomous analysis. This inconsistency is the result of lack of standardisation in preparation of biopsy slides. In order to avoid issues which could occur due to colour variation, we chose a target image from the glandular area of the image and applied its characteristics to all the patches of the training and testing dataset. There are number of stain normalisation techniques but we adopted the Reinhard stain normalisation method^31^ due to its time efficiency, available in our group’s Stain Normalisation Toolbox^32^. This stain normalisation approach uses a linear transformation to map the colour distribution of the source image to that of the target image. This is carried out by matching the mean and standard deviation of each channel of the source image to that of the target image in Lab colorspace. We also performed mean subtraction and scale normalisation to generate a training set of input patches with zero mean and unit norm.

#### Network Architecture

The network architecture consists of five downsampling layers and four upsampling layers. In each downsampling layer, the input is convolved with three filters: the first two of size 3 × 3 and the last one of size 1 × 1, all with stride 1. After convolution operations, 2 × 2 max pooling is performed with stride 2, allowing minimisation of the size of feature maps. In upsampling layers, as in UNET architecture, the input is first deconvolved with a filter, resulting in an increase in the size of the feature map, while reducing its depth (number of channels) by a factor of two. The resulting output is then concatenated with the center cropped feature maps from the corresponding downsampling layer followed by two 3 × 3 and one 1 × 1 size convolution operations. After the last upsampling layer, the output is convolved with a 1 × 1 filter to reduce the depth of feature map to the desired number of classes, which in our experiments is 2 (either gland or background). The whole network is trained using Adadelta to minimise the cross entropy loss function. Batch normalisation is performed on a mini-batch after each convolution operation. It is followed by an activation function, except for the last 1 × 1 convolution, before applying softmax. The addition of learnable bias is now ignored since the effect is compensated for by batch normalisation.

#### Segmentation Postprocessing

The softmax function at the end of the network produces a probability map, assigning to each pixel an estimate for the probabilities of belonging to a particular class. The probability map was thresholded to generate a binary map and the threshold value was selected empirically so that it separated the individual glands and generated an output map with few false detections. A number of morphological operations were performed to removed small objects, fill holes and separate slightly merged objects.

### Measuring Glandular Shape Aberrance

In order to measure the deformation of a gland, we define and calculate its *glandular aberrance* using the *BAM value*, also defined and calculated, as follows. Let *u* be a curve representing the glandular boundary.

**Figure 7:**
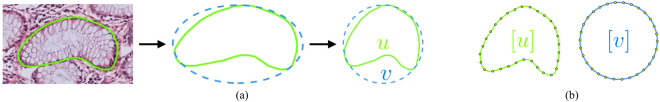
An illustration of the calculation of glandular aberrance. (**a**) Step 1: a closed curve representing glandular boundary *u* is extracted (green). Then, a minimum area enclosing ellipse *v* of *u* is calculated (dash blue). Next, the transformation that makes *v* become a circle is applied to *u*. (**b**) Step 2: shapes [*u*] and [*v*] are generated by scaling curves *u* and *v* such that they have unit path-length and the same number of representative points with zero mean.

**Step 1:** Let *v* be the minimum area ellipse enclosing *u*. Rescale *v* such that the lengths of its major and minor axes are equal. Apply the same transformation to *u*. Figure 7(a) illustrates this step.

**Step 2:** Rescale *u* and *v* such that both curves have unit path-length. Then move them so that their means (the centres of gravity of the curves) are at the origin. We also change the number of sampling points to that they are equal for the two curves. See Fig. 7(b).

**Step 3:** Calculate the BAM distance between the shapes of the two curves, as explained in the subsection below. The answer is the *BAM value* of a gland or the *glandular aberrance*.

The motivation behind these steps is to create a level playing field by comparing each glandular shape to a circle. Step 2 removes the the confounding effect of the angle at which one cuts through the tubular gland. After these steps are taken, cutting at a different angle of the section to the same gland would hardly alter the BAM value, if at all.

#### Best Alignment Metric (BAM)

Given two shapes for comparison, BAM operates on the principle of implicitly aligning the two shapes before computing the distance between them. We shall use ‘curve’ to refer to a particular instance of a parametrised closed curve in the plane and ‘shape’ to refer to an equivalence class of curves over the operations of translation, rotation and re-parametrisation. The BAM distance is defined between a pair of these equivalent classes. For given closed curves $u,\,v\in {C}^{\infty }(\mathrm{[0,}\,\mathrm{1],}{R}^{2})$, we denote the equivalence class of *u* by [*u*] and the equivalence class of *v* by [*v*]. We define $\hat{u}$ to be the curve whose mean is 0, obtained by translating *u* in the plane. Let *p*_*θ*_ be the planar rotation centred at the origin through an angle *θ*. We define the BAM distance as^11^

1 $${d}_{BAM}([u],[v])=\sqrt{{{\rm{\min }}}_{(r,\theta )}{\int }_{0}^{1}||\hat{v}(s)-{p}_{\theta }(\hat{u}(s+r)){||}^{2}ds},$$

where the argument (*s* + *r*) is taken modulo 1, and the minimum is taken over $\mathrm{[0,}\,\mathrm{1)}\times \mathrm{[0,}\,2\pi )$. In the rest of the paper, we will assume that all curves have their mean at the origin, so that $u=\hat{u}$.

In the discrete case, we assume that each curve *u* is represented by a cyclic sequence of *N* complex numbers, i.e., $u=\{{u}_{j}\in {\mathbb{C}}:j=\mathrm{0,}\,\cdots ,N-\mathrm{1\}}$, where points *u*_*j*_ must be equally spaced around the curve; in other words, $|{u}_{j+1}-{u}_{j}|$ is independent of *j* The BAM distance between discretely represented shapes is defined as

2 $${d}_{BAM}([u],[v])=\sqrt{\frac{1}{N}{{\rm{\min }}}_{(r,\theta )}\sum _{j=0}^{N-1}|{v}_{j}-{e}^{i\theta }({u}_{j+r}{)|}^{2}},$$

where the index (*j* + *r*) is taken modulo *N*, and the minimum is taken over $\{(r,\theta )\in \mathrm{\{0,}\,\cdots ,N-\mathrm{1\}}\times \mathrm{[0,2}\pi )\}$.

For a detailed mathematical description and fast computation of BAM and an approximation lemma on this metric, the reader is referred to the Supplementary Materials document.

### Glandular Aberrance Features

For each image, three features were calculated: the mean of the BAM values, the BAM entropy and the Regularity Index. The calculated BAM values corresponding to each gland in an image were observed to be in the range of 0 to 0.1. The mean of BAM values was computed by taking the average of BAM values corresponding to all the glands in an image. The second main feature, BAM entropy, is obtained by binning the BAM values for each image and assigning a probability to each bin. The number of bins was chosen using the minimum and maximum BAM values, calculated for all the glands in the dataset with a step of 0.015. As usual, empty bins did not contribute to the entropy. The third feature is obtained by taking the ratio of the number of glands in the first two bins to the total number of glands in the image. We call this ratio the *Regularity Index*. This ratio was considered to be useful for classification after examining the histograms of BAM values. For instance, if one looks at the top panel of Fig. 2, one sees that almost all the glands are very nearly circular or elliptical, resulting in small BAM values, mostly occupying first two bins. While for cancerous images (middle and bottom panel of Fig. 2), the BAM values are distributed in the bins of high BAM values. The efficacy of Regularity Index can be confirmed by its box plot, as shown in Supplementary Fig. 3 in the Supplementary Materials document. For a quantitative evaluation of this feature, we performed classification using two sets of features: **Feature Set 1** comprising average BAM value and BAM entropy and **Feature Set 2** comprising Regularity Index and Feature Set 1.

#### Feature Postprocessing

**Figure 8:**
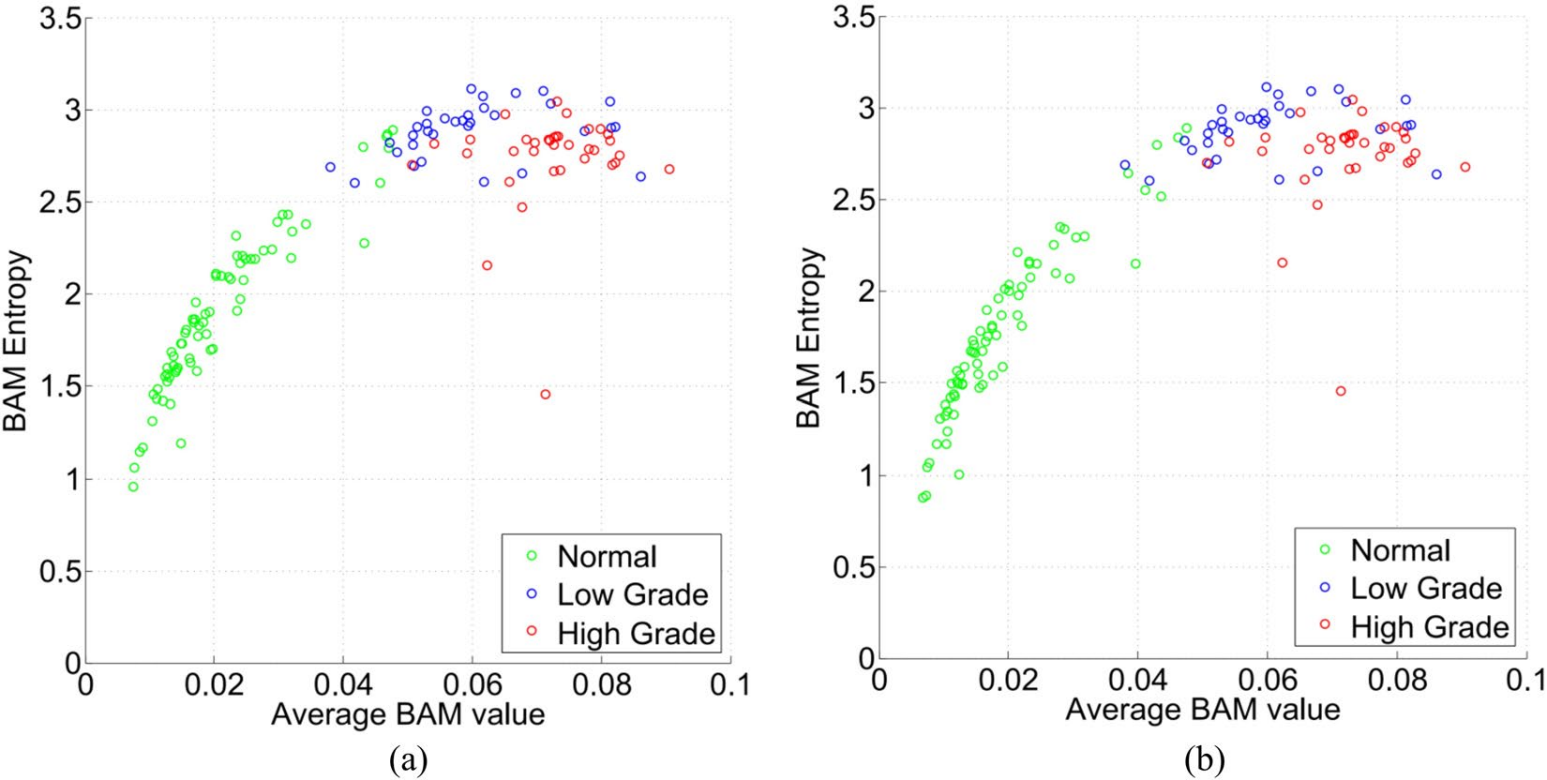
Scatter plots. (**a**) Scatter plot before feature postprocesing and (**b**) Scatter plot after feature postprocessing. Feature postprocessing involves removal of the BAM value of tangential sections of crypts and glands located at the image border but only for normal images.

**Figure 9:**
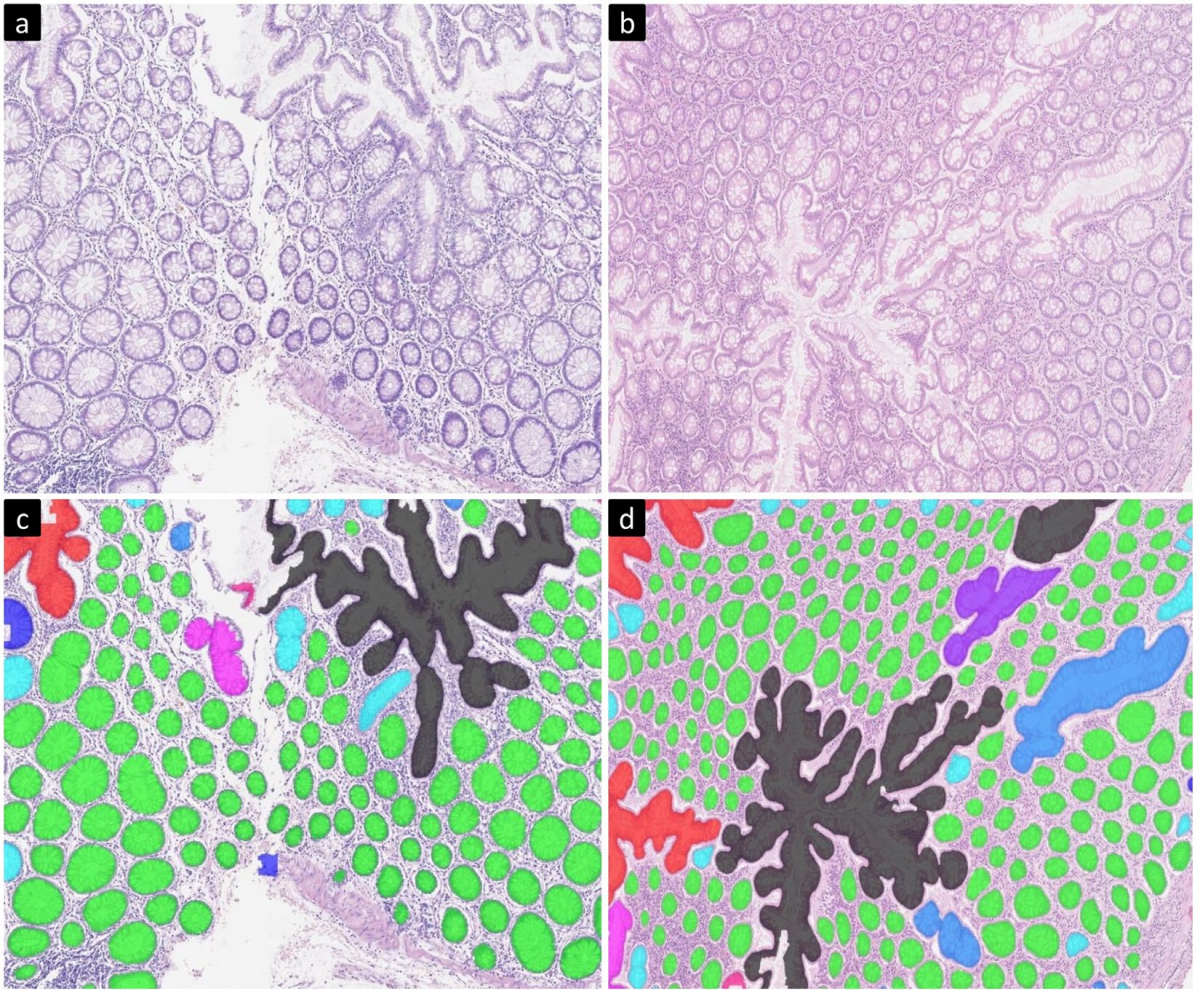
Patches taken from two different normal images. These images contain tangential sections of crypts, coloured with red and black, resulting in high average BAM values.

The scatter plot of average BAM values and BAM entropy is shown in Fig. 8(a). This plot showed unexpectedly high average BAM values and BAM entropy for normal images. On visual examination of normal images and BAM values overlaid on glandular objects, we observed some tangential sections of crypts (see Fig. 9). These tangential sections had very high BAM values giving rise to high average BAM value and BAM entropy for the image. We needed to remove these unusually large values for normal tissue, because they incorrectly indicated seriously diseased glands. Images with Regularity Index greater than a threshold value (selected empirically) were classified as normal images, enabling us to treat normal images slightly differently from cancerous images. We removed BAM values of tangential sections of crypts from images classified as normal, by applying area based thresholding (with threshold again selected empirically). A gland in a normal image can also have an unduly large BAM value if it crosses the boundary of an image, because part of the elliptical or circular shape has been cut off and discarded. Therefore glands located at the border of normal classified images were removed based on their location.

Scatter plots of average BAM values and BAM entropy before and after feature postprocessing are shown in Fig. 8. It can be seen that the average BAM and BAM entropy values are reduced after postprocessing resulting in an improvement in separation between normal and tumour as shown in Fig. 8(b).

### Tumour Grading/Classification

We performed two-class and three-class classification using our dataset in which each image was labelled/graded as normal, low grade or high grade. For two-class classification, normal vs tumour, we merged the low grade and high grade tumour images into one tumour class. The SVM classifier was trained to assign a grade to each image using both Feature Sets 1 and 2 to analyse their efficacy. We compared linear, polynomial, radial basis function (RBF) and sigmoid kernel SVM. We found that SVM with RBF kernel yielded the highest accuracy for three-class classification while for two-class classification, the performance of SVM with RBF and sigmoid kernel is comparable. The evaluation results presented in this paper are achieved using the RBF kernel SVM. The comparison results of different SVM kernels are shown in Supplementary Fig. 6.

## Electronic supplementary material

Supplementary Materials
