# Supplementary material for: Glandular Morphometrics for Objective Grading of Colorectal Adenocarcinoma Histology Images
Source: Sci Rep. 2017 Dec 4;7:16852. doi: 10.1038/s41598-017-16516-w (PMC5715083; doi:10.1038/s41598-017-16516-w)
Supplement: Supplementary file 1 — Supplementary Materials [file 41598_2017_16516_MOESM1_ESM.pdf]

Glandular Morphometrics for Objective Grading of Colorectal Adenocarcinoma Histology Images

Ruqayya Awan[1], Korsuk Sirinukunwattana[2], David Epstein[3], Samuel Jefferyes[2], Uvais Qidwai[1], Zia Aftab[4], Imaad Mujeeb[4], David Snead[5], and Nasir Rajpoot[2,5,*]

[1]Department of Computer Science and Engineering, Qatar University, Doha, Qatar
[2]Department of Computer Science, University of Warwick, Coventry, UK
[3]Mathematics Institute, University of Warwick, Coventry, UK
[4]Hamad Medical Corporation, Doha, Qatar
[5]Department of Pathology, University Hospitals Coventry and Warwickshire, Coventry, UK
[*]N.M.Rajpoot@warwick.ac.uk

Best Alignment Metric (BAM)

In this section we will explain what we mean by *shape space* and define and discuss the BAM metric on this space. Our theory works for rectifiable paths and in particular, for smooth paths. However we restrict our discussion to polygonal paths for the sake of simplicity. A boundary curve of a biological object in a micrograph will usually be given as a polygonal path, and a smooth path can be approximated by a polygonal path. Moreover complexity estimates for our algorithms present fewer technical difficulties if the inputs are polygonal.

Definition 1. A *polygonal simple closed curve* in the complex plane (abbreviation PSCC) is a map $u : [0,L] \to \mathbb{C}$ together with a cyclic sequence $(u_0, u_1, \ldots, u_{P-1}, u_P = u_0)$ of complex numbers, related to u as follows. The straight intervals $[u_{i-1}, u_i]$ are traversed successively, for $i = 1,2,\ldots,P$, by u at unit speed. We have $\sum_{i=1}^{P} |u_i - u_{i-1}| = L$, where L is the length of the curve. We assume that u has no self-intersections, except for the endpoints $u(0) = u(L) = u_0 = u_P$. We also assume that u goes in an anti-clockwise direction around the topological open disk that it bounds. We set $V(u) = P$. For $s \in \mathbb{R}$, we define $p_s : [0,L] \to [0,L]$ by $p_s(t) = s + t \mod L$. This is a convenient notation for changing the starting point of a parametrization of a simple closed curve.

Definition 2. By an *oriented shape* we mean an equivalence class of PSCCs u, defined by the following equivalence. Let u and v be PSCCs of the same length L. We say that u is *oriented shape equivalent* to v and write $u \sim v$ if there is an orientation preserving isometry g, and an $s \in \mathbb{R}$ such that $v = g \circ u \circ p_s$. (See Definition 1.) Our formal definition is that an *oriented shape* is an equivalence class of PSCCs under oriented shape equivalence. Since the lengths of two equivalent PSCCs are equal, it makes sense to talk of the *length* of (the boundary of) a shape. The reflection in a line of an oriented shape is also an oriented shape (possibly the same oriented shape, if it is symmetric under reflection), and we define an *unoriented shape* to be the union of an oriented shape and its reflection. Given a shape or an oriented shape, we can scale it to have length one.

For brevity, we will use the term "shape" in this paper to mean an oriented shape of length one. There is no need to consider unoriented shapes, because each of our shapes is compared with an associated ellipse, and an ellipse is invariant under reflection in either of its axes. Also we will not take scale into account.

Let σ_1 and σ_2 be two (oriented) shapes (of length one). We define

$$d_{\mathtt{BAM}}(\sigma_1, \sigma_2) = \inf\{d_2(f_1, f_2) \mid f_1 \in \sigma_1, f_2 \in \sigma_2\} \quad (1)$$

One can prove that this definition does indeed satisfy the standard axioms for a metric.

In order to have practical and fast computational methods, we approximate each length one PSCC by a cyclic sequence of complex numbers. The accuracy of the approximation is determined by N, the number of points in the cyclic sequence. We fix $N = 2^n$ for some positive integer n. (Later we will invoke Fast Fourier Transform (FFT) for fast computation, and this works best for powers of two.)

Definition 3. Given a PSCC $f : [0,1] \to \mathbb{C}$ of length one, we define its *N-approximation* $\mathtt{A}_\mathtt{N}(f)$ to be the cyclic sequence $(a_0, \ldots, a_N = a_0)$ of complex numbers given by $a_i = f(i/N)$. Note that this sequence is almost certainly not the same as the sequence of vertices used in the definition of the polygonal curve f. We have $\sum_{i=1}^{N} |a_i - a_{i-1}| \leq 1$, and, although the curve has length one, equality is rare. The union of the intervals $[a_{i-1}, a_i]$ may have self-intersections (though it will be a PSCC if N is large enough, depending on f). $\alpha(t) = a_0 = a_N$ to the

It is convenient to recast Definition 2 in the context of cyclic sequences.

Definition 4. By a *sequence shape* we mean an equivalence class of cyclic sequences of complex numbers, defined by the following equivalence. Let $A = (a_0, \ldots, a_N = a_0)$ and $B = (b_0, \ldots, b_N = b_0)$ be cyclic sequences of complex numbers of the same length $N = 2^m$. For $k \in \mathbb{Z}$, we define $B_k = (b_k, b_{k+1} \ldots, b_{k+N} = b_k)$, where the indices are taken mod N. We say that A is *(oriented) shape equivalent* to B and write $A \sim B$ if there is an orientation preserving isometry g, and a $k \in \mathbb{Z}$ such that $A = g(B_k)$.

In order to use N-approximations to estimate $d_{\mathtt{BAM}}$ between shapes, it is convenient to extend the definitions to cyclic sequences of complex numbers of the same length N. Let $A = (a_0, \ldots, a_N = a_0)$ and $B = (b_0, \ldots, b_N = b_0)$. We define

$$d_2(A,B) = \left(\frac{\sum_{i=1}^{N} |a_i - b_i|^2}{N} \right)^{1/2}. \tag{2}$$

The denominator N is usually omitted in defining the L^2 distance between two finite sequences. However, with this denominator, N-approximations converge as N tends to infinity.

We have the following result, which we state without proof.

Lemma 1. Let σ_1 and σ_2 be two shapes of length one. Let $f_1 \in \sigma_1$ and $f_2 \in \sigma_2$. There are euclidean translations t_1 and t_2 such that $A = t_1(\mathtt{A}_{\mathrm{N}}(f_1))$ and $B = t_2(\mathtt{A}_{\mathrm{N}}(f_2))$ each have mean 0. With B_k as in Definition 4,

$$\left| d_{\mathtt{BAM}}(\sigma_1, \sigma_2) - \inf_{1 \le k \le N, \theta \in [0,2\pi]} d_2(A\ ,\ e^{i\theta} B_k) \right| \le \frac{3}{2N}.$$

If $\alpha = (\alpha_1, \ldots, \alpha_N)$ and $\beta = (\beta_1, \ldots, \beta_N)$ are N-dimensional complex vectors, we recall the notation $\langle \alpha, \beta \rangle = \sum_{i=1}^{N} \overline{\alpha_i} \beta_i$ for the Hermitian inner product. We show how to minimize $d_2(A, e^{i\theta} B_k)$ in terms of the inner product. We write $\langle A, B_k \rangle = C_k \exp(i\psi_k)$, where $C_k \ge 0$. We want to find (k, θ) minimizing

$$\langle A - \exp(i\theta) B_k\ ,\ A - \exp(i\theta) B_k \rangle = \langle A, A \rangle + \langle B, B \rangle - 2\,\mathrm{Re}(\exp(i\theta) \langle A, B_k \rangle).$$

Equivalently we maximize $C_k\, \mathrm{Re}(e^{i(\theta + \psi_k)})$. This is done by choosing k so as to maximize $C_k = |\langle A, B_k \rangle|$ and then choosing

$$\theta = -\psi_k. \tag{3}$$

Fast Computation of BAM

The value of C_k as k varies can be computed by FFT with time complexity $O(N \log(N))$ as follows:

1. Compute $(f_j)_{j=0}^{N-1}$, the fast Fourier transform of $(\overline{a_j})_{j=0}^{N-1}$.
2. Compute $(g_j)_{j=0}^{N-1}$, the fast Fourier transform of $(b_{N-j-1})_{j=0}^{N-1}$.
3. Compute $(X_j)_{j=0}^{N-1}$, the inverse fast Fourier transform of $(f_j g_j)_{j=0}^{N-1}$.
4. Compute $k = \mathrm{argmax}_j |X_j|$. Then $C_k = |X_k|$ by the convolution theorem..

For $i = 1, 2$, σ_i is given by specifying a PSCC $f_i \in \sigma_i$. We can then determine A and B with time complexity $O(V(f_1) + V(f_2) + N)$, where $V(f_1)$ and $V(f_2)$ are the number of vertices used to define f_1 and f_2 respectively, as discussed in Definition 1. It follows that, if σ_1 and σ_2 are shapes of length one, with representative PSCCs f_1 and f_2, then $d_{\mathtt{BAM}}(\sigma_1, \sigma_2)$ is computed to within an error of $3/2N$ with time complexity $O(V(f_1) + V(f_2) + N \log(N))$. A reasonable scheme is to fix $N = 2^n$ greater than $V(f)$ for any PSCC f under consideration. We also choose N sufficiently large so that the error resulting from using N-approximations is small enough for one's purposes. This error is independent of f, as shown in Lemma 1.

Note that the above formulae do not consider the reflection $R(\sigma)$ of a shape σ in a line to automatically be considered to be the same shape as σ. However, in most biomedical applications, it would be very reasonable for us to change the definition of "shape" so as to make $R(\sigma) = \sigma$. It is a matter of chance which side of a tissue section will be uppermost when it is placed on a glass slide, and so, if σ is the shape of a gland in a micrograph, we would want $R(\sigma) = \sigma$. If we wish our shapes to be invariant under reflection, we change the definition of the distance between shapes to

$$\tilde{d}_{\mathtt{BAM}}(\sigma_1, \sigma_2) = \min\left(d_{\mathtt{BAM}}(\sigma_1, \sigma_2), d_{\mathtt{BAM}}(\sigma_1, R(\sigma_2))\right). \tag{4}$$

Another issue is whether or not to scale the shapes being analyzed. In many applications, we want our shapes to be scale invariant. This can be achieved by scaling the PSCCs to all have length one. If, on the other hand, one wishes to take length into account, we can define the square of the distance between a shape σ_1 of length λ_1 and a shape σ_2 of length λ_2 by

$$(d_{\mathtt{BAM}}(\sigma_1, \sigma_2))^2 = (\log(\lambda_1/\lambda_2))^2 + (d_{\mathtt{BAM}}(\sigma_1/\lambda_1\ ,\ \sigma_2/\lambda_2))^2.$$

One can prove that this extension satisfies the axioms for a metric on the space of unscaled oriented shapes.

BAM Comparisons

Comparison with Symmetric Difference and Fourier Descriptors

For comparison purpose, we experimented with the contours of human retinal pigment epithelial (RPE) cells. In this section, we compare BAM to two other approaches namely Symmetric Difference and Fourier Descriptors. Comparison is presented in terms of time taken to compute the shape distance and in terms of ability to generate five simulated shapes which closely resembles the original shape. For time comparison, we measured the total time taken to compute the distances of 100 pairs, dividing that number by 100 to give a measure of the time to compute the distance once. We ran this process 100 times to create a mean and standard deviation.

The Symmetric Difference Metric, denoted by d_{SD}, is defined on the measurable subsets of any measure space (X,μ), by the formula

$$d_{SD}(A,B) = \mu(A\Delta B) = \mu(A\setminus B) + \mu(B\setminus A).$$

This gives us a metric on regions with polygonal boundary by integrating the area over the symmetric difference. In order to apply the notion to shapes, we have to minimize $d_{SD}(A,\rho(B))$, where ρ varies over all orientation preserving isometries, that is, over all transformations resulting from translations and rotations. The speed and accuracy of this calculation will obviously depend on choices for the resolution of the binary images and the number of rotation angles considered. Even at this low resolution of 128×128 pixels and 10 rotation options, the computation time is already 1500 times slower than BAM. In the case of BAM, computations with different rotation angles is unnecessary, as shown by Equation 3.

With Fourier descriptors, a commonly used shape descriptor, we found that it is approximately 4.5 times faster than BAM. Here the accuracy depends both on the number of Fourier coefficients we decide to use, and on the number of rotation angles we wish to try, each of which affects the speed of computation linearly. Neither of these is relevant for BAM (see Equation 3), and so the factor 4.5 that we present here is somewhat artificial, as we can vary it up or down at our pleasure.

The average time taken with each of these three measures is shown in Supplementary Table 1. On comparing the simulated shape generated by Fourier descriptors and BAM, BAM outperforms Fourier descriptors, as shown in Supplementary Figure 1. This emphasizes the fact that aspects of shape information are lost when the phase information is removed and the power spectrum alone is not enough to faithfully represent shape. Symmetric difference, with the parameters we used, seems to be as good as BAM for selecting similar shapes, while being much slower.

	Best Alignment Metric	Symmetric Difference	Fourier Descriptors
Average Time	$35\pm1.6\mu s$	$53000\pm9200\mu s$	$7.8\pm5.4\mu s$

Supplementary Table 1. Time comparison of BAM with two shape difference measures: Symmetric difference and Fourier descriptors.

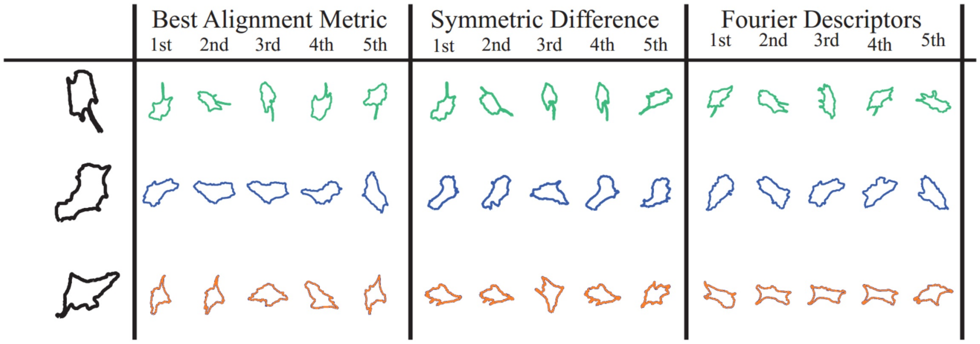

Supplementary Figure 1. Comparative performance of shape distance measures. Each row above shows a randomly selected target RPE cell outline, followed by 5 outlines identified as closest to the target outline (excluding outlines of the same cell as the target) according to 3 different shape distance measures.

Comparison with Square Root Elastic Measure

The Square Root Elastic (SRE) distance is highly regarded as a sensible measure of shape distance. SRE has a big advantage over BAM in the case of computer vision, because its elastic property enables it to follow changes in shape, as we might have

with a movie of a person running. When applied to images of tissue sections with no movement, this advantage disappears, and we are left only with the crippling disadvantage of slowness.

When computing distances between shapes, the choice of number of points around the curve (N) affects both accuracy and speed. To evaluate the performance of BAM and SRE in terms of accuracy and speed, five pairs of RPE cell curves were chosen. The BAM and SRE distances were computed between each pair with different number of sampling points. For each pair of shapes, an absolute relative error was computed and is presented in Supplementary Figure 2. It can be seen that error of the SRE measurements are not reliably below 1% until N = 1024.

The time taken to compute each shape distance with different number of N is shown in Supplementary Table 2. There is an exponential increase in computational time with SRE on increasing the number of sampling points while in case of BAM, the increase in computational time is linear.

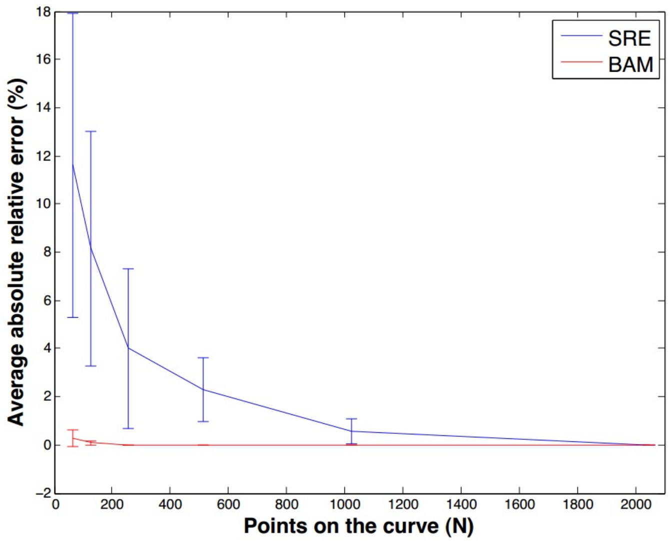

Supplementary Figure 2. Absolute relative error from curve sub-sampling. A plot of the mean absolute relative error (%) of SRE (blue) and BAM (red) as functions of N, the number of points taken around the curve. Error bars show standard deviation.

N	64	128	256	512	1024	2048
BAM time	5.0×10^{-3}	8.6×10^{-5}	9.1×10^{-5}	1.3×10^{-4}	1.8×10^{-4}	3.0×10^{-4}
SRE time	1.2	2.9	17	120	930	7400

Supplementary Table 2. A table displaying the average time (in seconds) taken to compute BAM and SRE distances. These distances are calculated for a range of values for N, which is the number of sample points at each curve.

Gland Segmentation

Network training

We made use of the open source library Tensorflow to train our segmentation network. The initial weights of the network were generated using a commonly followed approach, i.e., taking values randomly from a Gaussian distribution with standard deviation $\sqrt{(2/N)}$, where N is the total number of inputs to the computational unit. A batch size of 10 patches was used to train the network using a Dell T7610 computer with NVIDIA TitanX 12GB GPU. The network was trained with a dataset of more than 120,000 patches and the training was stopped after 1,44,000 iterations (12 epochs). We used weighted cross entropy as a cost function. This penalises at each pixel according to the deviation of its network-calculated probability from 1. A weighted map was generated, assigning a large weight at the dilated gland boundaries (slightly overlapped between background and gland around the boundaries), so that the network learned the boundary of each gland.

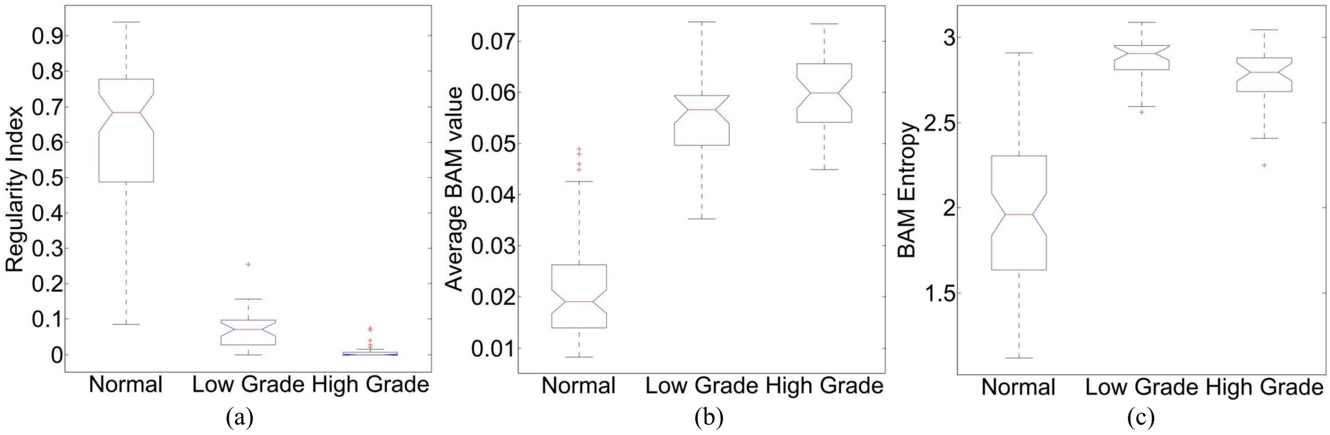

Supplementary Figure 3. Box plots of (a) Ratio of frequency of first two bins of BAM to the overall frequency, referred as the *Regularity Index*, (b) Average BAM and (c) BAM entropy. Note all these box plots are generated before postprocessing to remove the BAM values of tangential sections of crypt and glands at image borders for normal images.

Method	F1score				Object.Dice				Object.Hausdorff				Rank sum
	Part A		Part B		Part A		Part B		Part A		Part B		
	Score	Rank	Score	Rank	Score	Rank	Score	Rank	Score	Rank	Score	Rank	
CUMedVision2	0.912	1	0.716	3	0.897	1	0.781	4	45.418	1	160.347	5	15
Our Approach	0.879	4	0.775	1	0.879	4	0.823	1	62.812	5	114.964	1	16
ExB1	0.891	3	0.703	4	0.882	3	0.786	2	57.413	4	145.575	2	18
ExB3	0.896	2	0.719	2	0.886	2	0.765	5	57.350	3	159.873	4	18
Freiburg2	0.870	5	0.695	5	0.876	5	0.786	3	57.093	2	148.463	3	23

Supplementary Table 3. Comparison of our segmentation algorithm with top four participants of GlaS challenge. The evaluation is carried out according to the challenge criteria. The overall performance is given by rank sum and is calculated by adding the ranks obtained in all three measures. The entries are listed in a descending order based on their rank sum.

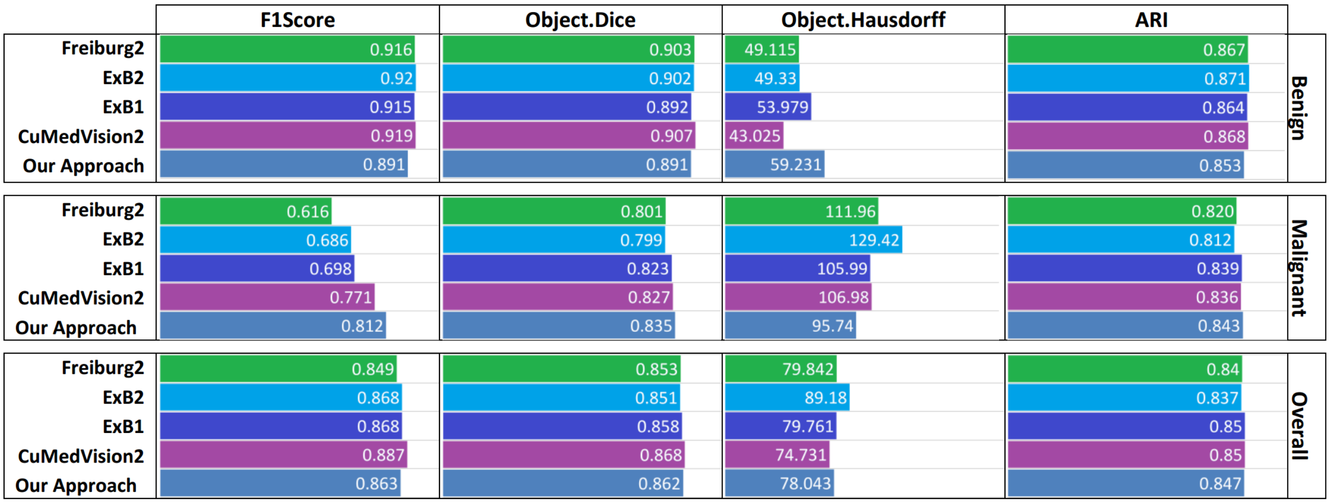

Supplementary Figure 4. Comparison results of our approach with the top four participants of GlaS challenge using four evaluation measures. The results are computed for three sets of test images: normal, malignant and the combination of normal and malignant images. For the malignant test set, our approach outperforms all other programs. ARI stands for adjusted rand index.

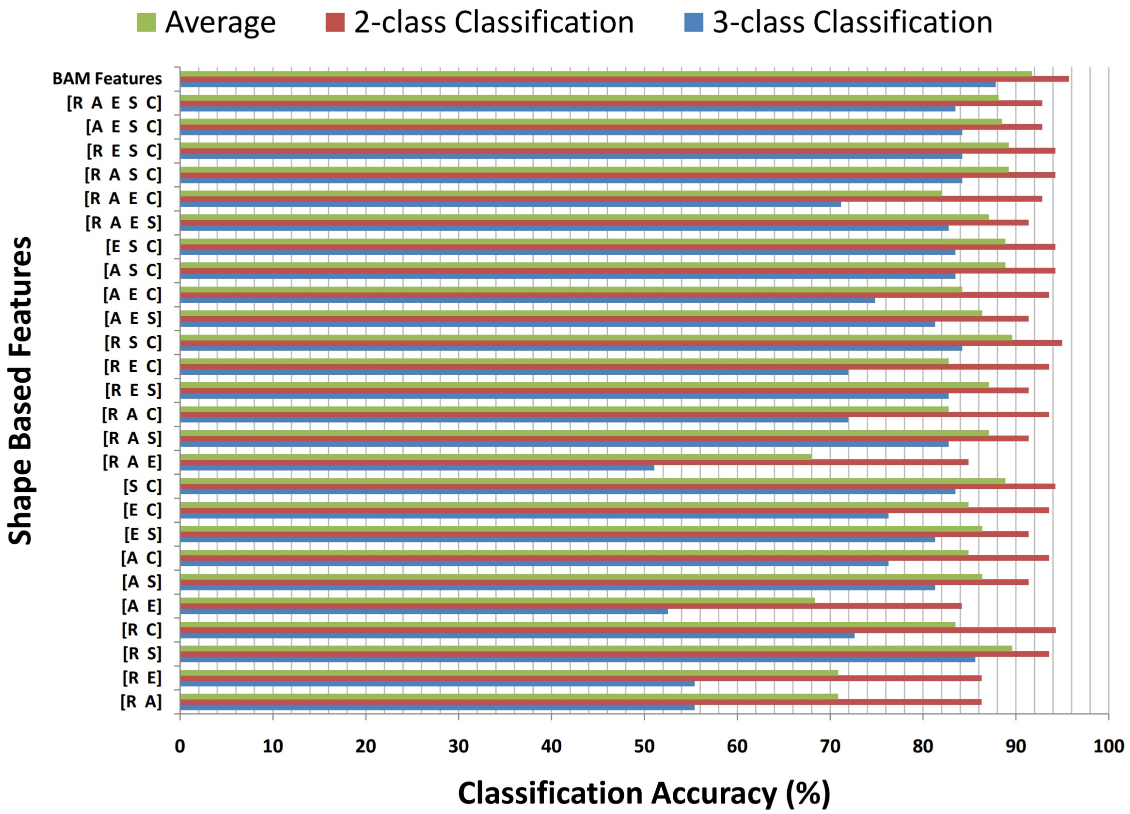

Supplementary Figure 5. Comparison results of our proposed BAM features with combination of relevant shape based features. R, A, E, S and C represent roundness, aspect ratio, elongation, solidity and convexity respectively. The average accuracy represents mean of accuracy obtained for 2-class and 3-class classification. Linear SVM when trained with BAM features outperformed other SVM classifiers trained with combination of other shape features. Maximum average accuracy is obtained with [R A S C] and [R A E C] and is equal to 89.23% for both feature combinations while accuracy obtained with BAM is 91.74%

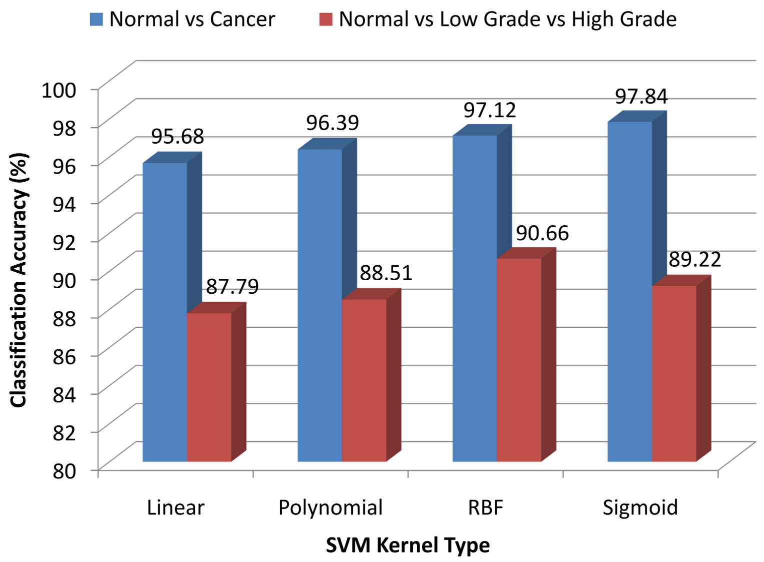

Supplementary Figure 6. Comparative performance using four different kernels with SVM for two and three class classification.

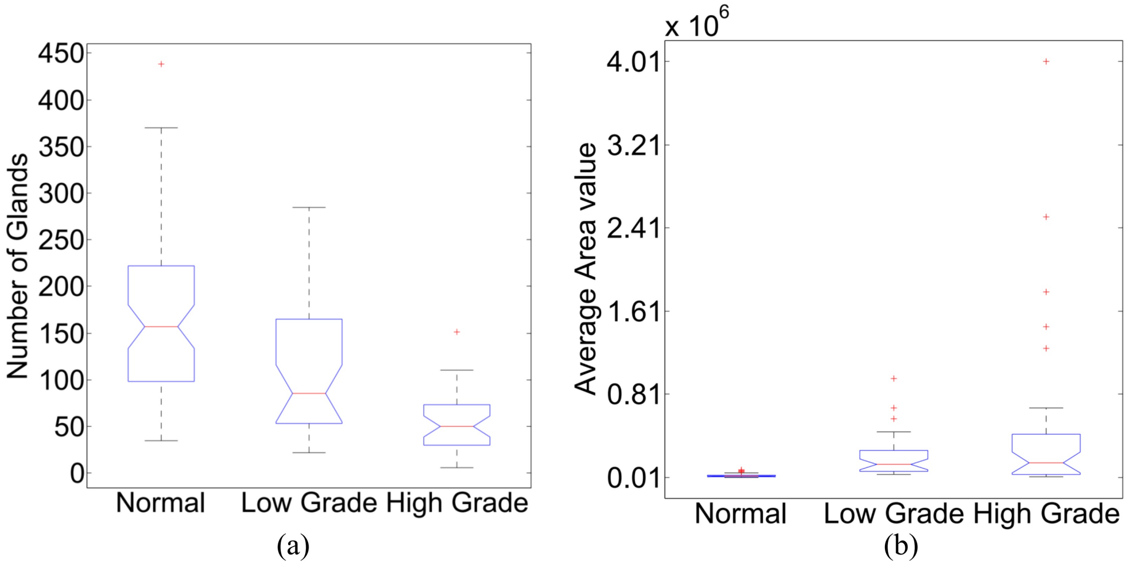

Supplementary Figure 7. Box plots of (a) Total number of glands, (b) Average area of glands. An overlap can be seen among all three classes which makes these features inappropriate for classification.

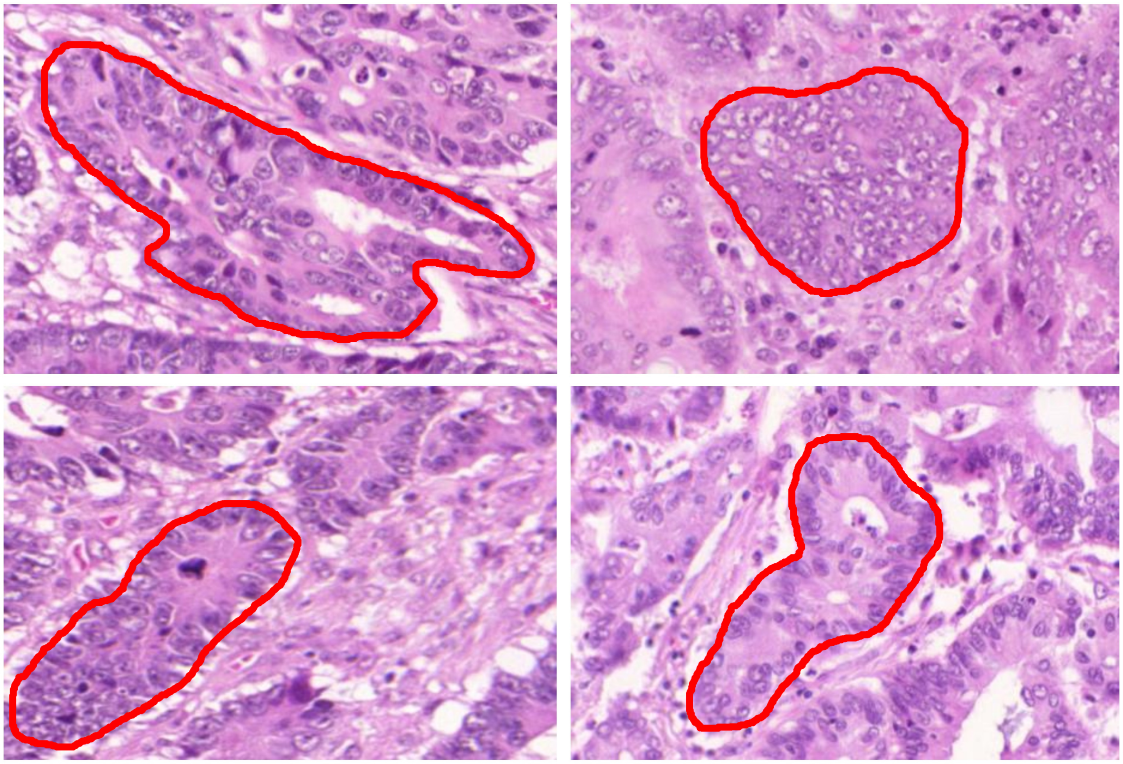

Supplementary Figure 8. Example images of glands (delineated with red marker) with not much lumen. Due to the absence of lumen or the inner structure of gland particularly in high grade cancerous tissues, the circumscribed boundary of glandular structures is considered rather than inscribed minimal convex polygon.
